# Proteomics-Based Identification of Dysregulated Proteins and Biomarker Discovery in Invasive Ductal Carcinoma, the Most Common Breast Cancer Subtype

**DOI:** 10.3390/proteomes11020013

**Published:** 2023-04-03

**Authors:** Anca-Narcisa Neagu, Danielle Whitham, Logan Seymour, Norman Haaker, Isabella Pelkey, Costel C. Darie

**Affiliations:** 1 Laboratory of Animal Histology, Faculty of Biology, “Alexandru Ioan Cuza” University of Iasi, Carol I bvd. No. 20A, 700505 Iasi, Romania; 2 Biochemistry & Proteomics Laboratories, Department of Chemistry and Biomolecular Science, Clarkson University, Potsdam, NY 13699-5810, USA

**Keywords:** breast cancer, IDC, DCIS-to-IDC progression, proteomics, dysregulated proteins, dysregulated pathways, biomarker discovery

## Abstract

Invasive ductal carcinoma (IDC) is the most common histological subtype of malignant breast cancer (BC), and accounts for 70–80% of all invasive BCs. IDC demonstrates great heterogeneity in clinical and histopathological characteristics, prognoses, treatment strategies, gene expressions, and proteomic profiles. Significant proteomic determinants of the progression from intraductal pre-invasive malignant lesions of the breast, which characterize a ductal carcinoma in situ (DCIS), to IDC, are still poorly identified, validated, and clinically applied. In the era of “6P” medicine, it remains a great challenge to determine which patients should be over-treated versus which need to be actively monitored without aggressive treatment. The major difficulties for designating DCIS to IDC progression may be solved by understanding the integrated genomic, transcriptomic, and proteomic bases of invasion. In this review, we showed that multiple proteomics-based techniques, such as LC–MS/MS, MALDI-ToF MS, SELDI-ToF-MS, MALDI-ToF/ToF MS, MALDI-MSI or MasSpec Pen, applied to in-tissue, off-tissue, BC cell lines and liquid biopsies, improve the diagnosis of IDC, as well as its prognosis and treatment monitoring. Classic proteomics strategies that allow the identification of dysregulated protein expressions, biological processes, and interrelated pathway analyses based on aberrant protein–protein interaction (PPI) networks have been improved to perform non-invasive/minimally invasive biomarker detection of early-stage IDC. Thus, in modern surgical oncology, highly sensitive, rapid, and accurate MS-based detection has been coupled with “proteome point sampling” methods that allow for proteomic profiling by in vivo “proteome point characterization”, or by minimal tissue removal, for ex vivo accurate differentiation and delimitation of IDC. For the detection of low-molecular-weight proteins and protein fragments in bodily fluids, LC–MS/MS and MALDI-MS techniques may be coupled to enrich and capture methods which allow for the identification of early-stage IDC protein biomarkers that were previously invisible for MS-based techniques. Moreover, the detection and characterization of protein isoforms, including posttranslational modifications of proteins (PTMs), is also essential to emphasize specific molecular mechanisms, and to assure the early-stage detection of IDC of the breast.

## 1. Introduction

Worldwide, invasive ductal carcinoma (IDC) and invasive lobular carcinoma (ILC) are the major histological types of invasive breast cancer (IBC) among women of different races [1]. IDC, also called infiltrating ductal carcinoma, is the most common histological subtype of breast cancer, with an incidence of approximately 80% of all diagnosed BCs in women of any age [2,3], and 85% of all male breast cancers [4]. IDCs could be considered as a group of tumors [5] that demonstrate great heterogeneity in clinical characteristics, treatment management, prognoses, as well as in gene expression [6] and intra-tumor morphology or biomolecular landscape [7].

Ductal carcinoma in situ (DCIS), a subtype of BC where atypical epithelial cells are restricted to the milk ducts, is considered a non-invasive or pre-invasive as well as a non-obligate precursor to IDC [8,9]. An important proportion of patients with BC emphasize the co-existence of IDC with DCIS (IDC-DCIS) [10]; women with DCIS experience higher long-term risk of IDC and death from BC than women in the general population [11]. However, many women with DCIS will probably never progress to IDC [8], so that it remains a great challenge to determine which patients should be over-treated, or which need to be monitored without aggressive therapy [12]. Considering that the molecular continuum from normal duct to IDC, passing through atypical ductal hyperplasia (ADH) and/or DCIS, is still not well understood [8], the finding of novel molecular biomarkers of BC progression is absolutely necessary for developing prediction models to avoid the underestimation of IDC [13] and, consequently, to assure more adequate patient diagnosis and treatment [9].

The DCIS progression to IDC has been explored by a large variety of studies, including animal model investigations [14] as well as other model-based studies [15], biopsy specimen review [13], population-based screening programs [11], and DCIS surveillance trials [16]. The role of intra-tumor heterogeneity in the progression of DCIS to IDC has been demonstrated in the context of four evolutionary models: independent lineage, convergent phenotype, evolutionary bottleneck, and multiclonal invasion [17]. The contribution of genetic/epigenetic aberrations and tumor microenvironment (TME) features to the progression from DCIS to IDC has been demonstrated and reviewed [14,18,19]. Furthermore, a progressive loss in basal layer integrity heading towards IDC, coupled with two epithelial–mesenchymal transitions (EMT), has been associated with the progression of DCIS to IDC [8]. A plethora of potential biomarkers of DCIS progression to IDC have been discovered using genomics- [20,21], epigenomics- [22], transcriptomics- [8,9], proteomics- [3], interactomics- [23], and metabolomics- [24] based specific techniques. Several hallmarks of breast cancer progression towards IDC have been described: sustainability of proliferative signaling, genomic instability and mutation, resisting cell death, replicative immortality, evading growth suppressors, energy metabolism rewiring, inducing angiogenesis, tumor promoting inflammation, evading immune destruction, and invasion-metastasis cascade [25]. Each of these hallmarks was defined by a specific proteomic profile characterized by dysregulated proteins, pathways, and biological processes. Moreover, there are molecular features associated with DCIS to IDC progression that emphasize subtype specificity, as well as molecular features present in some DCIS lesions that may be considered a predictor of disease progression [19]. Although the tumor genome and transcriptome are important fields for the discovery of novel biomarkers, the dysregulated proteome expression reflects more accurately the essential changes in cancer pathophysiology [26]. Proteomics-based techniques, such as LC–MS/MS, MALDI-ToF MS, SELDI-ToF-MS, MALDI-ToF/ToF MS, MALDI-MSI, or MasSpec Pen, applied to in-tissue, off-tissue, BC cell lines and liquid biopsies, improve the diagnosis of IDC, its prognosis, and treatment monitoring. Programs of EMT and EMT-related pathways, as well as proteomic remodeling of TME, are deeply involved in IDC. Future proteomics-based studies may be focused on non- or minimally-invasive biomarker discovery using liquid biopsies, as well as on investigation of protein isoforms for isoform-based diagnoses [27], post-translational modifications (PTMs), and protein–protein interaction (PPI) networks, with high specificity for differentiating IDC from other breast pathologies.

## 2. Differentiating IDC from Other BCs

IDC refers to the neoplastic proliferation and microinvasion of luminal epithelial cells into surrounding breast stroma, by passage through the ductal wall [5], following the disruption of the ductal basement membrane and myoepithelial cell layer. Based on the histological proprieties of tumors, several subtypes of IDC have been described [28]: the classical nonspecific subtype/not otherwise specified subtype (IDC-NST/IDC-NOS) [5], breast invasive apocrine carcinoma (BAC) [29], medullary carcinoma of the breast (MBC) [30], mucinous carcinoma/colloid carcinoma (MCB) [31], invasive papillary carcinoma (IPC), invasive micropapillary carcinoma (IMPC), and tubular ductal carcinoma (TC). Synthetically, IDCs can be classified as ”no special type” because these tumors do not emphasize sufficient morphological characteristics to be classified as a distinct histological type, and ”special type” that present specific cellular and molecular landscapes [32]. However, there were studies which showed that IDC and MBC are completely independent and different types of breast malignancies [33]. Undoubtedly, the most common histological type of BC is the invasive breast carcinoma of no special/nonspecific type [6], constituting about 40–75% of all invasive breast carcinomas [32]. Many studies analyzed the molecular patterns of BC so that, based on comprehensive gene expression profiles patterns, four clinically relevant molecular BC subtypes have been described: luminal A, luminal B, enriched HER2 (HER2+), and triple-negative (TNBC) [32]. These molecular patterns are specific for both IDC and ILC.

It is possible, but not mandatory, for IDC to develop from high-grade ductal carcinoma in situ (DCIS) lesions, which are pre-malignant epithelial proliferations that are confined to a lactiferous duct [34]. Histologically, the pure DCIS is surrounded by an intact myoepithelial cell layer, with no signs of invasion within the basement membrane. The immunohistochemical detection of several biomarkers for myoepithelial cells, such as CD10/neprilysin/membrane metallo-endopeptidase [35], smooth muscle actin (SMA), calponin (CLP), and p63 [36], confirm the diagnosis of pure DCIS, while the gradual loss of these myoepithelial cell differentiation markers indicates a compromised myoepithelium, and suggests DCIS progression to invasive disease [34]. DCIS lesions may consist of multiple clones of tumor cells [34], harboring specific genetic and/or epigenetic aberrations that may progress to life-threatening IDC with varying metastatic potential, if left untreated [18]. Studies indicate that 12–40% of these pre-invasive intraductal lesions progress to invasive disease, if untreated [18,35]. In the early stages of the invasive disease, small groups of epithelial tumor cells become adjacent to DCIS lesions, such that DCIS and invasive cancer tissues are present in the same lesion. In 21.3–76.9% of cases, DCIS lesions co-exist with IDC, the IDC-DCIS representing a disease phenotype that is different from pure IDC [10]. In these cases, the identification of main characteristics that may predict DCIS progression to IDC would be of clinical importance [14]. Molecular studies showed that synchronous DCIS and IDC may be remarkably similar [18,35]. A study published by Moelans et al. concluded that there were no significant differences between DCIS and IDC, suggesting that DCIS is genetically as advanced as its invasive counterpart [37]. However, different histological grades of DCIS have been associated with distinct genomic landscapes that progress to IDC following different pathways [37]. Thus, the IDC-associated DCIS was assessed to be more aggressive than pure DCIS at a genomic level, and, consequently, it should be potentially considered IDC [38]. To avoid unnecessary aggressive treatments that affect many women diagnosed with DCIS that could evolve into IDC, is imperatively necessary to identify and validate new protein biomarkers and pathways able to differentiate DCIS pre-invasive lesions from those which may progress to IDC [15].

On the other hand, gene expression profiling has revealed distinct patterns among ”typical” ILCs and IDCs, while the ”ductal-like” ILCs closely resemble IDCs in their transcription patterns [1]. Moreover, invasive ductal carcinoma with lobular features (IDC-L) overexpresses E-cadherin immunostaining, which confirms its ductal origin and may make it considered to be a variant of IDC; meanwhile, the clinical and biological characteristics are more similar to that of ILC [39]. Even if IDC and ILC are treated as a single entity in clinical trials, the molecular differences between ILC and IDC may have important therapeutical implications [40]. For example, it was demonstrated that luminal ILC had worse outcomes that luminal IDC. Hence, different treatment strategies should be used for luminal ILC than for luminal IDC [41].

## 3. Models of the Malignant Continuum from DCIS to IDC

Four models have been proposed to explain the progression of DCIS towards IDC [34,42]. Thus, the independent lineage/evolution model shows that both DCIS and IDC develop in parallel and independent from each other, evolving from distinct cancer-initiating cells/clonal cell populations. In this case, the initiating cell lineages do not share same mutations or copy number aberrations (CNAs) [17]. The convergent phenotype model proposes that DCIS of different genotypes progresses to form IDC of the same phenotype. The direct evolutionary bottleneck model sustains that multiple individual subclones characterized by multiple somatic mutations are present in DCIS, only one of which may escape from the duct and progress to IDC. The multiclonal invasion model shows that multiple DCIS clones can escape from the duct, migrate, invade, and persist into surrounding tissues to establish invasive carcinomas. The multiclonal invasion model was identified by topographic single-cell sequencing (TSCS) that revealed a direct genomic link between DCIS and IDC subpopulations, with the genomic instability and mutation evolving in ducts prior to invasion [43]. There are findings that demonstrate that genetically unrelated DCIS and IDC can co-occur in the same breast, supporting the evidence that DCIS is a non-obligate precursor of IDC [44]. The applicability of these evolutionary models may vary from patient to patient, so that the biomarkers of DCIS to IDC progression should be correlated with intra-lesion genetic heterogeneity and putative mechanisms of BC progression [44].

## 4. Molecular Biomarker Discovery and Related Technological Advancements

Rapid and recent advances in molecular profiling technologies have generated extensive biomolecular data in genomics, epigenomics, transcriptomics, proteomics, interactomics, and metabolomics fields, emphasizing novel aspects of BC biology at multiple levels of multi-omics interaction networks [45]. Next generation sequencing (NGS), single-cell sequencing (SCS) and mass spectrometry imaging (MSI) are the most innovative technologies that have great potential to provide new insights into the transition from DCIS to IDC [17].

In the genomics field, whole-exome sequencing (WES) of DCIS and IDC has shown high similarities in copy number profiles between these two breast pathologies [20], emphasizing that DCIS that progresses to IDC displays a pattern of clonal selection, and harbors higher levels of intra-lesion genetic heterogeneity than DCIS where no clonal selection was observed [44]. Single-cell genetic analysis of DCIS and IDC also revealed high tumor heterogeneity yet conserved genomic imbalances and gain of *MYC* during progression [21]. Using a gene expression profiling-based study, Dettogni et al. emphasized three genes (*FGF2*, *GAS1*, and *SFRP1*) as potential biomarkers for the transition of stationary to migrating invasive epithelial cells [9]. Epigenomics-based studies showed that DNA methylation alterations are early events in the DCIS to IDC progression, and may represent valuable biomarkers that predict invasive recurrence more accurately than classic measures of DCIS progression [22]. Transcriptomics, based on RT-qPCR, validated progression-associated transcripts, such as mRNA that mediate transition from DCIS to IDC, emphasizes that MMP11 and COL10A1 characterize pure DCIS with a high risk of developing into IDC [46]. Furthermore, gene expression signatures of DCIS lesions, identified by RNA-seq-identified processes and biomarkers, were associated with progression towards IDC [8]. Transcriptomics studies based on single-cell RNA sequencing (scRNAseq) identified several tumor and TME features associated with DCIS progression, and provided genomic-associated signatures with DCIS-to-IDC pathobiology [14]. Metabolomics-based analyses, using liquid chromatography multiple reaction monitoring mass spectrometry (LC-MRS/MS) and untargeted gas chromatography mass spectrometry (GC-MS), have been successfully applied for the identification of metabolic alterations in tissue and serum samples of patients with IDC [24].

Even if the genomic classification of BC subtypes has made remarkable advances in BC diagnosis and prognosis [47], clinical assessment and newer classifications are also based on protein expression profiling [48]. For example, the breast cancer classification based on proteotypes obtained by sequential windowed acquisition of all theoretical fragment ion spectra (SWATH) mass spectrometry (MS) established key proteins for BC subtype classification [49]. There are two distinct approaches of classic proteomics analysis that showed complementary abilities for the detection of cancer-specific aberrations at the peptide and proteoform levels, and for measuring the differential expressions of proteins and proteoforms [50]: shotgun/bottom-up/peptide-centric approach, and top-down/protein-centric approach. Between them, the recently developed middle-down proteomics covers the analysis of middle-range peptides (3–10 kDa) [51]. Bottom-up proteomics is useful for identifying thousands of proteins in complex samples, and is based on the use of proteases, such as trypsin, before the resulting small peptide (0.7–3 kDa) detection, separation by liquid chromatography (LC), and sequencing using tandem mass spectrometry (MS/MS). Furthermore, protein mixtures may be separated by electrophoresis, and then individual proteins are digested and analyzed using matrix-assisted laser desorption/ionization (MALDI)-MS in a method called peptide mass fingerprinting (PMF) [52]. Bottom-up proteomics can be used for the identification of a peptide, protein, and PTMs in a peptide/protein, as well as for quantitative proteomics [52]. Top-down proteomics is useful for the direct analysis of small- to medium-sized intact proteins (10–30 kDa), and also enables the analysis of proteoforms, such as PTMs, at the intact protein level. Thus, intact/whole proteins, or a mixture of proteins, are analyzed for molecular mass in MS mode and further fragmented to provide partial fragments in MS/MS mode [52]. Middle-down proteomics also uses protein digestion, and allows for better protein coverage, including isoform identification [51].

In order to assess the expression profile of the DCIS proteome, as well as the expression profile of invasive biomarkers, isobaric tag for relative and absolute quantitation (iTRAQ) technology coupled with nLC–MS/MS analysis has been successfully used [53]. Based on the proteomic signature across the BC cell models, the LC–MS/MS technique revealed a stage-specific reprogrammed metabolism [54]. HER2 overexpression has been reported in the case of DCIS tumors that progress to IDC [55]. Moreover, HER2-interacting partners, such as junctional adhesion molecule-A (JAM-A), which regulates HER2 expression, have been identified as overexpressed in aggressive DCIS lesions, in correlation with angiogenetic and apoptotic pathway alterations [56].

To avoid invasive tumor tissue biopsies or surgeries, over the last decades, various omics-based strategies led to significant advances in searching for non-invasive or minimal-invasive biomarkers for all-stage as well as early-stage BC diagnoses in cancer liquid biopsies. To improve the identification of low-molecular-weight or low-abundance proteins and protein fragments that exist in bodily fluids in very low concentrations and are “invisible” to shotgun proteomics, sample preparation may be engineered to capture and enrich this special part of the proteome. Thus, core-shell hydrogel nanoparticles (HNs) are able to capture low-molecular-weight proteins and peptides with high affinity by baits immobilized in the core, allowing 10,000-fold amplification of the analyte concentration [57]. Consequently, the LC–MS/MS technique may identify new IDC protein biomarkers or emphasize accurate IDC-specific protein signatures [3]. Moreover, to reveal the serum-based protein profiling of IDC patients by MALDI-ToF MS, magnetic bead-based weak cation exchange chromatography (MB-WCX) and immobilized metal ion affinity chromatography (MB-IMAC-Cu) purification methods allow for capturing low-abundance proteins or peptides, which distinguishes patients with early-stage IDC from healthy individuals [58]. Consequently, these enrichment-based methods coupled with MS may lead to the identification of robust blood-based molecular signatures of IDC, consisting of a single protein or panel of proteins, for the validation of clinically accessible blood-based tests to support/confirm the mammography-based BC screening [3].

Recently, for in-tissue proteomics-based biomarker detection, a highly sensitive MS-based approach called single-pot, solid-phase-enhanced, sample preparation-clinical tissue proteomics (SP3-CTB) has been used to perform the comprehensive quantification of protein expression; it utilizes archived formalin-fixed paraffin-embedded (FFPE) BC surgical specimens to characterize the heterogeneity of BC at the protein level in a clinically-applicable manner, and to identify putative biomarkers for existing immunotherapies [48]. In surgical oncology, modern approaches should have a better ability to perform sensitive, rapid, and accurate “proteome point sampling”, as well as “proteome point characterization” of biological tissues for breast cancer profiling and identification of breast cancer types or subtypes. MS-based technology is also used for the molecular intraoperative characterization of healthy and tumor tissues only in a few seconds. For example, a non-destructive sampling technique merges a handheld and biocompatible device, the MasSpec Pen, which is connected to a mass spectrometer, to discriminate the proteomic profiles of normal breast and lymph node, IDC tissue, and IDC metastasis to lymph node, in order to detect the residual invasive disease at the tumor margin [59]. Moreover, two spatially targeted MS analysis optimized workflows have recently been reported that use a human BC model: the first one is applicable for thin-slice analysis, and uses transmission-polarized light imaging (polarimetry)-guided desorption electrospray ionization mass spectrometry imaging (DESI-MSI) with histological validation; the second one explores a polarimetry-guided MS platform for thick tissue assessment by developing reflection-mode polarimetric imaging coupled with a handheld picosecond infrared laser (PIRL) MS ablation probe that requires minimal tissue removal/invasive biopsies [60], and preserves intact proteins from tissues without changing their conformation, PTMs/proteoforms, or enzyme activity [61]. These recently reported methods should be analyzed and considered for rapid and accurate ex vivo and/or in vivo MS profiling that allows for the accurate differentiation and delimitation of tissue types in IDC.

## 5. Proteomics-Based Investigation of Dysregulated Proteins, Processes, and Pathways in IDC

**Figure 1:**
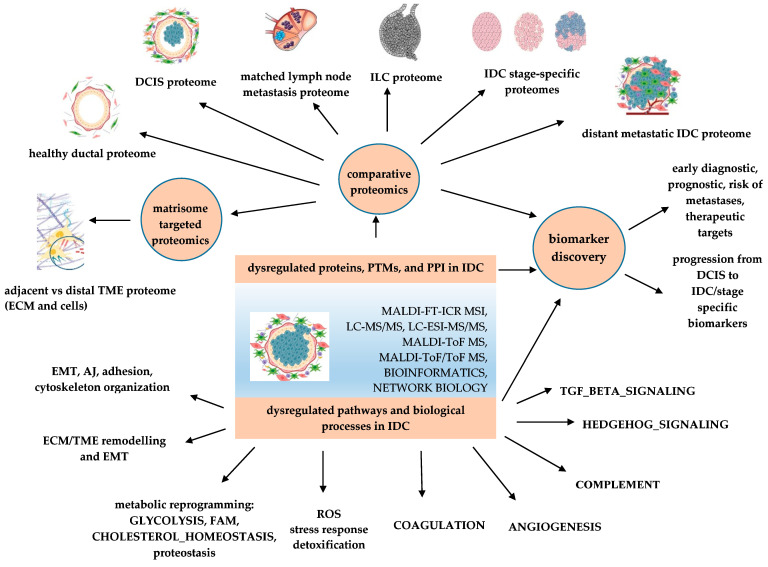
Applications of proteomics-based identification of dysregulated pathways and biomarker discovery in IDC. PTMs—post translational modifications; PPI—protein-protein interaction; AJ—apical junction; DCIS—ductal carcinoma in situ; ECM—extracellular matrix; EMT—epithelial–mesenchymal transition; FAM—fatty acid metabolism; IDC—invasive ductal carcinoma, ILC—invasive lobular carcinoma; ROS—reactive oxygen species; TME—tumor microenvironment.

Direct IDC tissue proteomics, as well as off-tissue, non- or minimally invasive liquid biopsies and BC cell lines proteomics-based analysis has been reported as related to IDC (Table 1 and Figure 1). Liquid chromatography tandem mass spectrometry (LC–MS/MS), matrix-assisted laser desorption/ionization time-of-flight MS (MALDI-ToF-MS), surface-enhanced laser desorption/ionization time-of-flight MS (SELDI-ToF MS), MALDI ToF tandem mass spectrometry (MALDI-ToF/ToF MS), MALDI-Fourier transform ion cyclotron resonance (MALDI-FT-ICR), mass spectrometry imaging (MSI), as well as MasSpec Pen, have all been used as classic or advanced techniques to perform proteome profiling of IDC in various experiments. These techniques have been used to distinguish IDC from healthy controls by analyzing solid tumor samples [62,63,64]; interstitial fluid and primary tumor cell culture [65]; serum [3,58,66,67]; tear fluid [68,69]; nipple aspirate fluid (NAF)/ductal lavage fluid (DLF) [69,70,71,72,73]; urine [74]; saliva [75]; and milk [76,77,78,79] These methods can also differentiate healthy or benign breast disease vs. lymph node ± IDC vs. matched lymph node metastases (LNM) ex-vivo [80,81], in vivo [59], and in serum [82]. Different stages of IDC, including early-stage/early-detection vs. normal breast tissue [83]; IDC vs. ILC tissues [84]; IDC tumor-adjacent stroma vs. tumor-distal stroma [85]; cancer-associated fibroblasts (CAFs)-related proteins in IDC-NST vs. ILC [86]; tumor-associated macrophages (TAMs)-related proteins in IDC vs. healthy controls [27]; IDC vs. other cancers (ovarian, prostate, lung, colon cancers, melanoma, and cytosarcoma phyllodes) vs. healthy controls by analysis of various body fluids, such as salivary proteome [75], serum proteome [3], and interstitial fluid, as well as cultured primary cell proteomes [65]; IDC-targeted matrisome proteins, such as MMPs, in IDC vs. non-tumoral extracellular matrix (ECM) [87,88]. Moreover, proteomics-based techniques are useful for novel candidate biomarker discovery for the early-stage detection of IDC [3], for proteomic profiling of different IDC subtypes such as ER+/HER2/*neu* [23], or for interrelated aberrant pathways analysis and PPI networks [89].

The dysregulated proteins detected by proteomics approaches in IDC tissue are encountered in different cellular compartments of cancer cells, such as in the plasma membrane [82], cell junctions [84], cell projections, cytoplasm [82], cytoskeleton [62,80], endoplasmic reticulum, ECM [87], Golgi apparatus, lysosomes, ribosomes and proteasome [81], microtubules, nuclei [82], and endosomes/secreted proteins [70]. Both the MALDI-ToF and LC–MS/MS techniques are able to identify protein alterations in infiltrating carcinomas of the breast, including glycolytic enzymes, molecular chaperones, cytoskeletal-related proteins, antioxidant enzymes, immune and inflammation-related proteins [63] with various molecular functions, such as structural molecules, enzyme regulators, transcription regulators, regulators of catalytic activities, and signal transducers [82].

### 5.1. Programs of EMT and EMT-Related Pathways Are Deeply Involved in IDC

It is well known that both genetic and proteomic intra-tumor heterogeneity, as well as biomolecular and histological characteristics of the tumor microenvironment, play central roles in the progression of DCIS to IDC [18]. This progression is often associated with gene expression programs of epithelial-to-mesenchymal transition (EMT) and myoepithelial cell-specific genes that are overexpressed in invasive cancer compared to pure DCIS [95]. LC–MS/MS [85,87], LC–MS^E^ and MALDI-MS/MS [23], MALDI-ToF MSI [96], MALDI-ToF/ToF MS, and MALDI FT-ICR-MSI-based proteomics [94] identified and quantified a plethora of biomarkers within tumor cells, as well as in their associated ECM, which are deeply involved in the EMT process.

First of all, the EMT-related markers in IDC are cytoskeletal proteins belonging to the actin cytoskeleton, the microtubule network, and the intermediate filaments or cytoskeletal-associated proteins involved in motility mechanisms [62] and/or proteins involved in desmoplastic reaction/ECM remodeling [97], such as actin isoforms (ACTB, ACTG), tubulin isoforms (TUBB, TUBA1A, TUBA1B), vimentin (VIM), tropomyosin isoforms (TPM4), keratins (KRT19, KRT8), filamin isoforms (FLNA), talin (TLN1), tenascin (TNC), integrin (ITGA2B), transgelin (TAGLN), profilin (PFN1), collagen isoforms (COL1A1, COL1A2, COL14A1), thrombospondin (THBS2), decorin (DCN), periostin (POSTN), mimecan/osteoglycin (OGN), fibronectin (FN1), and metalloproteinases (MMP-2, MMP-9) (Table 2).

The EMT process is considered to be the key crossroad between metabolism and tumor progression [115]. The EMT pathway is deeply associated with metabolic reprogramming [116] to promote and sustain motile and aggressive cells involved in tumor progression. HALLMARK_GLYCOLYSIS has been identified as the primary bioenergetics pathway involved in cell motility and cytoskeletal remodeling in BC, among other tumor types [117]. Proteomics techniques highlighted a plethora of metabolic-related enzymes in IDC cells, such as PGK1, PK, GAPDH, TPI, FBP, ENO1/ENO2, and ALDOA. Furthermore, proteomics techniques with connected bioinformatics approaches emphasized the potential links between EMT and other dysregulated pathways involved in cancer progression, such as HALLMARK_COAGULATION, that may provide the EMT-engaged CTCs with enhanced colonizing proprieties [89,118]; HALLMARK_IMMUNE RESPONSE that may be regulated by EMT programming [74,119]; HALLMARK_COMPLEMENT that participates in mediating EMT in multiple tumor tissues and models [70,120]; HALLMARK_ANGIOGENESIS [89] that cooperates with vasculogenesis, chemotaxis, and coagulation in BC-related invasion [121]; protein homeostasis alteration into a global context of remodeling invasive cancer tissue homeostasis based on the downregulation of DNA repair proteins, upregulation of ribosomal, lysosomal, and proteasomal proteins; elevated rates of protein translation, deregulation of protein folding machinery followed by accumulation of unfolded proteins [81] and deregulated chaperonins [62]; HALLMARK_REACTIVE_OXYGEN_SPECIES_PATHWAY [80] is known to induce EMT, glycolytic switch, and mitochondrial repression in BC cells [122] by several overexpressed enzymes, such as PRDX3/4/6, SOD1/SOD2, and GPX-1/4, identified via UHPLC-EASY spray ionization source [81] or HSP27, HSP20, HSP70, and HSPB1 molecular chaperones, identified by a LC–MS^E^, MALDI-MS/MS proteomics-based approach in ER+/HER2/*neu* negative subtype of IDC [23]. Thus, understanding the aberrant pathways involved in EMT may provide essential insights that lead to protein biomarkers and therapeutic target discovery in pre-invasive and invasive BC [123]. To explore IDC metabolism at the proteome level, MALDI-ToF/ToF was used to emphasize that glycerol-3-phosphate dehydrogenase 1 (GPD1) and monoacylglycerol lipase (MAGL) involved in triacylglyceride metabolism were downregulated in BC tissue in comparison to healthy counterparts, signifying that these enzymes might be promising tissue-based protein biomarkers with predictive value for BC [90].

### 5.2. Proteomic Remodeling of Tumor Microenvironment (TME) Is One of the Most Important Hallmarks of IDC

The TME consists of cellular components (i.e., fibroblasts, endothelial cells, immune cells, adipocytes) and non-cellular components (i.e., fibrillar collagen and other ECM proteins, growth factors, cytokines). In the epithelial cells of invasive breast cancer, the genes and proteins involved in synthesis and organization of the ECM have been detected as significantly overexpressed [124]. Thus, the TME disruption based on ECM remodeling and stiffening [87,125], dysregulation of stromal cell interactions, and aberrant gene/protein expression in stromal and/or myoepithelial cells are linked to the progression of DCIS to IDC [42]. The proteomic analysis of ECM (Table 3) may lead to cancer biomarkers discovery, which offers an increased potential for an accurate prognostic of pathological processes towards a predictive and personalized therapy [126]. Thus, a targeted matrisome analysis based on both liquid chromatography-selected reaction monitoring (LC-SRM) and liquid chromatography-data dependent acquisition (DDA) tandem mass spectrometry (LC–MS/MS), identified several ECM proteins, such as *COL12A1*, *THBS-2*, *FN*, and *TNC*, which have lower expression levels in normal breast tissue, but are overexpressed and co-localized within the disorganized stromal compartment in IDC tissue [87]. Furthermore, another proteomic study based on two-dimensional gel electrophoresis (2-DE) coupled with MALDI-ToF MS, emphasized that MMP-2 and MMP-9 matrix metalloproteinases are primarily responsible for basement membrane and peri-cellular ECM rearrangement [88]. Both MALDI-FT-ICR and mass spectrometry imaging approaches, named ECM-IMS, and high-resolution accurate mass (HRAM) nanoLC-ESI–MS/MS techniques were used for the investigation of TME proteomic heterogeneity into a tissue microarray (TMA) that included different breast pathologies, such as inflammation, hyperplasia, fibroadenoma, IDC, and ILC compared with normal adjacent tumor tissue, emphasizing a heterogeneous collagen type environment and other ECM-associated proteins in the central tumor [94].

Modern top-down and bottom-up MS-based proteomic techniques, especially those based on MALDI-ToF MS and LC–MS/MS, allowed for understanding of proteomic differences between cancer-associated fibroblasts (CAFs) and their normal fibroblast counterpart; the metabolic reprogramming associated with fibroblast activation; the reciprocal metabolic cross-talk between CAFs and cancer cells that involves the identification of CAF-derived proteins which act as regulators of cancer cell proliferation as well as the contribute to the CAFs secretome; these represent some among a long list of other ECM proteins that interact or remodel ECM, which leads to a complex proteomic profile of tumor matrisome [127,128]. To demonstrate the role of cancer-associated adipocytes (CAAs) in breast cancer cell migration, invasion, and resistance to therapy, DEPs in BC cells co-cultured with CAAs isolated from human breast adipose tissue have been identified and quantified using iTRAQ labelling and LC–MS/MS [129]. Pathway analysis demonstrated that CAAs emphasized a paracrine role in the enrichment of proteins involved in metabolism, ubiquitin proteasome, and purine synthesis.

**Table 3:** IDC-dysregulated proteins involved in ECM/TME remodeling and EMT.

Dysregulated Proteins	Genes	Proteomics-Based Methods	Functions	Associated Roles in Cancer	References
Tenascin	*TNC*	LC–MS/MS	ECM protein	partial EMT marker [130]; cell adhesion, tissue remodeling, transduction of cellular signaling pathways [131]	[87]
Collagen isoforms	*COL1A1*, *COL1A2*, *COL14A1*	LC–MS/MS; MALDI-FT-ICR MSI; HRAM, nanoLC-ESI–MS/MS	TME/ECM protein	cancer fibrosis, EMT [132,133]	[85,94]
Fibronectin	*FN1*	LC–MS/MS	component of the mammary mesenchymal compartment of breast tumor	cell invasion, metastasis, tumor progression, EMT [134]	[85]
Periostin	*POSTN*/*OSF-2*	FFPE, LCM, IHC, RT-PCR; LC–MS/MS	secreted ECM cell adhesion glycoprotein	EMT, proliferation, adhesion, migration [135]	[136]
Thrombospondins	*THBS1*/*TSP1*, *THBS2*/*TSP2*	LC–MS/MS	ECM proteins	cell adhesion, invasion, migration, proliferation, apoptosis, tumor immunity [137]	[85,87]
Decorin	*DCN*	LC–MS/MS	small leucine-rich ECM proteoglycan	overexpression decreases migration, invasion, stemness and tumor growth and metastasis [138]	[85]
Lumican	*LUM*	LC–MS^E^, MALDI-MS/MS	small leucine-rich ECM proteoglycan	EMT regulator [139]	[23]
Mimecan/osteoglycin	*OGN*	LC–MS/MS	small leucine-rich ECM proteoglycan	inhibits BC cell proliferation and reverses EMT via repressing PI3K/AKT/mTOR pathway [140]	[85]
Matrix metalloproteinases	*MMP-2*, *MMP-9*	2-DE, MALDI-ToF MS	Zn-dependent endopeptidases	ECM remodeling, tumor initiation, progression, metastasis [141]	[88]

Abbreviations: COL1A1—collagen type I alpha 1 chain; COL1A2—collagen type I alpha 2 chain; COL14A1—collagen type XIV alpha 1 chain; DCN—decorin; ECT/TME—extracellular matrix/tumor microenvironment; EMT—epithelial-mesenchymal transition; FN1—fibronectin 1; HRAM-MS—high-resolution, accurate-mass spectrometry; LC-MS^E^—liquid chromatography mass spectrometry in data-independent analysis mode; LC-MS/MS—liquid chromatography tandem mass spectrometry; LUM—lumican; MALDI-MS/MS—matrix-assisted laser desorption/ionization tandem mass spectrometry; MMP-2—metalloproteinase 2; MMP-9—metalloproteinase 9; OGN—osteoglycin; POSTN/OSF-2—periostin/osteoblast specific factor 2; RT-PCR—reverse transcription polymerase chain reaction; THBS1/TSP1—thrombospondin 1; THBS2/TSP2—thrombospondin 2; TNC-tenascin C.

### 5.3. Proteomics-Based Investigation of the Breast Cancer Proteomic Continuum Concept (BCPCC) in IDC for Non-Invasive Biomarker Discovery

In a previous published paper [142], we emphasized the central role of proteomics in characterization of the breast cancer cell continuum concept (BCCCC) that integrates the heterogeneous populations of neoplastic and cancer-associated cells into a continuum from the tumor initiation moment in breast ductal epithelium towards the colonization of distant metastatic niches in various tissues, via circulating tumor cell populations (CTCs). The BCCCC is sustained by a breast cancer proteomic continuum concept (BCPCC), where each phenotype of neoplastic and tumor-associated cells, as well as their microenvironments, are characterized by an adaptive proteomic profile that may be assessed in solid tissues, cell lysates, and liquid biopsies by complex proteomic approaches. Thus, both BCCCC and BCPCC allow for understanding of the tumorigenic cascade based on the analysis of cellular and non-cellular players involved in cancer progression, DEPs and/or accurate biomarkers, biological processes and multiple pathways, from the moment when a tumor arises in the mammary ductal epithelium towards metastasis-related events in distant organs.

To avoid invasive tissue biopsies or surgeries, over the last few decades, proteomics strategies allowed for significant advances in searching for non-invasive or minimally-invasive biomarkers for early-stage BC diagnosis, exploring the proteomes in liquid biopsies, such as blood and blood-derivatives [3], NAF/DFL, milk, urine, saliva, sweat, tears fluid, or breath [143]. Circulating proteins, consisting of blood proteome and cancer secretome that can be detected in measurable amounts in blood, as well as proteins present in other bodily fluids, may be used for the determination of disease risk, early diagnostics, treatment monitoring, prognostication, and for the assessment of disease progression [144]. Enzyme-linked immunosorbent assay (ELISA), MS, antibody array and aptamer-based proteomics allow for the detection of hundreds or thousands of proteins [144]. The identification of low molecular-weight (LMW) proteins and protein fragments in blood, which may be captured and enriched by advanced sample preparation technologies engineered coupled with LC–MS/MS, such as those using hydrogel nanoparticles (HNs), may lead to the identification of robust blood-based molecular signatures of BC; these signatures consist of a single protein or a panel of proteins for the validation of clinically accessible blood-based tests to support/confirm the mammography-based BC screening [3].

For example, galectin-binding protein/galactoside-binding soluble 3 binding protein (*LGALS3BP*/*GAL3BP*/*LG3BP*/*90K/Mac-2BP*) is a large, multitask-secreted, and hyperglycosylated 90 kDa protein that is expressed in the majority of human cells [145], including epithelial cells in breast and tear ducts, as well as in cancer cells [68]. This protein was first identified as a cancer- and metastasis-associated protein, being overexpressed in cancer-associated extracellular vesicles (EVs), also emphasizing an intracellular role in the innate immune response [145]. GAL3BP induces galectin-mediated tumor cell aggregation to increase the survival of cancer cells in the blood stream during the metastatic process [146], and inhibits monocyte-derived fibrocyte differentiation, blocking the formation of the fibrous sheet around the tumor, and allowing tumor cells to invade into the surrounding stroma [147]. GAL3BP also induces vascular endothelial growth factor in human BC cells, and promotes angiogenesis [148]. In BC biopsies, the overexpression of cancer cell-associated LGALS3BP was detected at the edges of tumors, where the cancer cells invade the surrounding stroma [147]. Hence, following the BCPCC, GAL3BP was present in cancer cells, was secreted in the ECM or tumor cell medium [149], and was also detected at high levels in serum and other bodily fluids, such as tears [68], saliva, urine, semen [150], proximal fluid [151], and the milk [78] of patients with different cancers, including IDC [146]. A comparative proteomic study analyzed the in vitro progression of BC based on LC–MS/MS identified GAL3BP as a highly secreted protein in tumorigenic/locally invasive MCF10 and tumorigenic/metastatic MCF10CA BC cell lines, and found that it was undetected in non-tumorigenic MCF10A and premalignant/tumorigenic MCF10AT cell lines [152]. Moreover, an LC–MS approach was applied to determine the sequences of N-glycans on GAL3BP from MCF7 and MDA-MB-231 cells, especially the sequences with terminal sialylation and fucosylation, in order to explain its role in cancer cell aggregation and metastasis [146]. Finally, an LC–MS/MS technique identified the overexpression of GAL3BP in the tear fluid [68] of breast cancer patients, as well as in the proximal fluid of several BC cell lines [151].

Following the same pattern of BCPCC, a plethora of proteins have been detected as invasive biomarkers in primary breast tumors, as well as non-invasive biomarkers in liquid biopsies, using different proteomic approaches. Vimentin (VIM), a protein used as a mesenchymal biomarker that acts as a central player in EMT processes, was detected via MALDI-ToF/ToF MS to be overexpressed in IDC PBT compared to its low levels in matched lymph node metastases (LNM) [80], in IDC compared with ILC samples [84], as well as in fresh frozen (FF) breast tissue biopsies of ER+/HER2/*neu* negative IDC, using LC–MS^E^ and MALDI-MS/MS [23]. Vimentin was also detected at high levels in the sera of patients with IDC, where the vimentin gene was also found to be hypomethylated [153]. MALDI-ToF MS also quantified a vimentin DNA methylation process in breast tumors and matched control pairs [154].

Tenascin (TNC) has been identified as a dysregulated protein in human milk, using combinatorial electrophoresis and LC–MS/MS-based proteomics [78], as well as in the aligned collagen stroma of invasive breast carcinoma using a matrisome-targeted analysis also based on LC–MS/MS [85,87]. Calcium binding proteins involved in signaling pathways, such as annexins and several members of the S100 family of proteins, have been found to be dysregulated in human milk, using combinatorial electrophoresis and LC–MS/MS-based proteomics [78]; in tears, using LC–MS/MS [68]; as well as in FF breast tissue biopsies of ER+/HER2/*neu* negative IDC, using LC–MS^E^ and MALDI-MS/MS [23]. Aberrant expression levels and/or glycosylation modification related to abnormal biological characteristics of glycoprotein alpha-1-antichymotrypsin (AACT) have been reported in tumors, including IDC tissue, which suggests that AACT may serve as novel biomarker for tumor diagnosis and prognosis [155]. AACT has been detected by protein profiling of the serum [67,82] and milk [78] of IDC patients, using LC–MS/MS and MALDI-ToF MS [67].

### 5.4. Proteomics-Based Investigation of Protein Isoforms in IDC

The clinical relevance of protein isoforms in tumorigenesis, as well as in cancer diagnosis, prognosis, and treatment, is becoming increasingly evident. The detection and characterization of protein isoforms are essential to emphasize molecular mechanisms, and to ensure the early detection of BC [156]. Isoform-based quantitative data allow for better cancer patient stratification with diagnostic values [27], whilst the isoform-specific changes in the BC proteome may offer an explanation for the distinct phenotypic proprieties of tumor cells during BC progression [157]. Furthermore, the isoform-specific peptides are known to distinguish normal breast tissue from BC [156]. To perform protein isoform detection and quantification, the development and validation of LC–MS/MS-based targeted proteomics assays represent an alternative method for WB and IHC that often lack specificity, simultaneous detection ability of multiple isoforms, and reproducibility [27]. Proteomics-based studies of various BC cell lines, tissue samples, and liquid biopsies highlighted the importance of protein isoforms in the characterization of non-invasive vs. different invasive carcinoma types, such as ILC and IDC (Table 4). Different isoforms may be produced from alternative splicing (AS), single-nucleotide polymorphisms (SNPs), and posttranslational modifications (PTMs) [156]. An LC–MS/MS-based targeted proteomics assay resulted in the simultaneous and accurate quantification of biological samples for two major isoforms of the folate receptor (FR) family. The membrane-associated proteins FRα and FRβ showed that the overexpression of FRα was detected in BC cells and tissue samples, while FRβ was overexpressed in tumor-associated macrophages (TAMs) but not in epithelial cells [27]. A differential quantitative proteomic analysis based on SDS-PAGE and HPLC–MS/MS was also able to characterize the alteration of progesterone receptor isoforms’ A and B ratios (PRA/PRB) during BC progression in the context of the altered BC proteomes involved in cell metabolism, proliferation, and apoptosis [157]. High-throughput plasma proteomics profiling in BC allowed for the identification of novel biomarkers, which are AS isoforms [158]. Serum protein profiling using 2-DE separation coupled to MALDI-MS may be used as technique for the exploration of protein alterations in patients with IDC [67]. Thus far, four isoforms of haptoglobin precursor and two isoforms of alpha-1-antitrypsin precursor (α1-AT) were upregulated in the sera of patients with IDC with various tumor stages in comparison to healthy women [67]. α1-AT overexpression has also been detected in FFPE sections of breast tumors using IHC [67]. Nevertheless, the integration of MS-based proteomics with next-generation sequencing, also called proteogenomics, allows for deciphering the heterogeneity of BC based on the quantification of proteins and PTMs [159].

## 6. Conclusions and Future Perspectives

Invasive ductal carcinoma (IDC) is the most common histological subtype of malignant breast cancer (BC), and accounts for 70–80% of all invasive BCs. The significant protein biomarkers of the progression from DCIS to IDC are still poorly identified, validated, and clinically applied. Thus, in precision oncology, it is a great challenge to determine which patients should be over-treated versus which need to be actively monitored without aggressive treatment.

Direct IDC tissue-based proteomics applied to FF and FFPE tissue samples, non- or minimally invasive liquid biopsies, and BC cell lines proteomics-based analyses, has been reported to be related to the detection of dysregulated proteins, biological processes, and pathways that drive IDC development and progression. LC–MS/MS, MALDI-ToF-MS, SELDI-ToF MS, MALDI-ToF/ToF/MS/MS, MALDI-FT-ICR MSI, as well as MasSpec Pen technologies, have been identified as useful for proteomics-based detection of characteristic protein profiles in IDC; they are able to differentiate between DCIS vs. IDC, as well as between ILC and IDC that are currently similarly treated in clinical practice. We emphasized that programs of epithelial–mesenchymal transition (EMT) and EMT-related pathways, such as those involved in adhesion, metabolism reprogramming, TME remodeling, immune response, coagulation, complement and reactive oxygen species pathways are the most important hallmarks of IDC, and may be deeply analyzed and further exploited for the identification of new panels of proteins and candidate biomarkers for IDC. To avoid invasive tissue biopsies or surgeries for direct IDC tissue-based proteomics, the molecular strategies may converge in the search for non-invasive or minimally invasive biomarkers for early-stage BC diagnosis. Proteomic profiles of blood and blood-derivatives, interstitial fluid, NAF/DFL, milk, urine, saliva, sweat, tears fluid, or exhaled breath may be used for innovative diagnostic assays; they may serve as starting points for advanced technologies, such as lab-on-chips for rapid, point-of-care detection and early diagnosis of IDC. Proteomics-based studies successfully complete the comprehensive genomics, transcriptomics, and metabolomics studies of IDC. Furthermore, the molecular characterization of IDC contributes to the discovery of novel targets for drug development and targeted therapies.

In modern surgical oncology, for the ability to perform sensitive, rapid, and accurate “proteome point sampling” and “proteome point characterization” in biological tissues for BC profiling and for the identification of cancer types or subtypes, MS-based technology should be the method of choice. MS-based technology is also used for the molecular intraoperative characterization of healthy and tumor tissue only in a few seconds, based on modern sampling techniques, such as handled MasSpec Pen or PIRL-DESI MSI, which have the ability to perform in vivo proteomics-based analyses or involve minimal tissue removal. To improve the identification of low-molecular-weight (LMW) or low-abundance proteins and protein fragments that exist in bodily fluids in very low concentrations and are “invisible” to shotgun proteomics, sample preparation techniques may be engineered to capture and enrich this part of the proteome. However, few studies have used proteomics-based analyses of IDC-associated proteoforms in breast primary tumors or liquid biopsies, even if top-down proteomics could reveal significant differences between ductal non-invasive and invasive breast cancer tissues, as well as significant differentially expressed intact proteoforms with a biomarker value. These approaches should be analyzed and taken into account for rapid and sensitive ex vivo and/or in vivo MS profiling, for the accurate differentiation and delimitation of tissue types in IDC.

## Figures and Tables

**Table 1:** Clinical relevance of proteomics-based investigation of dysregulated pathways and biological processes involved in IDC.

Relevance	Biological Samples	Other Conventional Analytical and Coupled Methods	MS-Based Proteomics	Results	Dysregulated Pathways and BP	References
Comparison of IDC vs. healthy counterparts; comparison of IDC vs. cytosarcoma phyllodes	interstitial fluid, primary cell culture	FC, IF, WB	TmT, HPLC–MS/MS + MudPIT	DEPs, quantitative proteomic profile	EMT; IFγ pathway; cell invasion, motility, survival, adhesion, cell cycle/proliferation, Wnt signaling, proteasome, and apoptosis	[65]
	FF	2-DE	MALDI-ToF MS	activity levels of MMP-2 and MMP-9 much higher in IDC	ECM remodeling	[88]
	FF	2-DE, DIGE	MALDI-ToF/ToF	proteome profiling	triacylglyceride (TAG) metabolism	[90]
Comparison of IDC vs. ILC	IDC and ILC tissue samples	2-DE	MALDI-ToF/ToF MS	proteome profiling	AJ; EMT; GLYCOLYSIS	[84]
	FFPE ILC and IDC-NST tissue samples	TMA, H&E, IHC	-	comparison of CAFs-related proteins	cell growth, angiogenesis, macrophage recruitment, ECM remodeling	[86]
Comparison of IDC vs. other cancers (ovarian, lung, prostate, colon cancers, and melanoma) vs. healthy controls	saliva	WB, IHC	nLC–MS/MS	DEPs; PPI	cell-motility related proteins, cytoskeletal organization, ECM remodeling	[75]
	serum	nanoparticle-based protein enrichment technology	LC–MS/MS	IDC-specific protein signatures	candidate biomarkers	[3]
Comparison of IDC vs. healthy/benign controls	tissue samples	2D SDS-PAGE; IHC	LC–MS/MS; MALDI-ToF-MS	DEPs and candidate biomarkers	deregulated chaperonins, stress-related proteins, cytoskeletal proteins involved in motility mechanisms, metabolic enzymes, immunologic responses	[62,63,64]
	serum	magnetic bead-based serum fractionation	MALDI-ToF MS	serum protein profiling	metabolic enzymes and protease activity as biomarkers for diagnosis and drug development; protein isoforms detection	[58,66,67]
		nanoparticle-based protein enrichment technology	LC–MS/MS	LMW and protein fragments; IDC-specific protein signatures; early-stage IDC ECM biomarkers	EMT/migration, cell proliferation, adhesion and metastasis	[3]
	milk	1D-SDS-PAGE, 2D-PAGE	nLC–MS/MS	protein profiling, DEPs	putative biomarkers	[76,77,78,79]
	tear fluid	1D-SDS-PAGE, ELISA	SELDI-ToF MS; MALDI-ToF/ToF; LC–MS/MS	biomarkers for early detection	metabolic reprogramming; immune response	[68,69,91]
Comparison of bilateral matched pair NAF/DLF proteomes in unilateral IDC	NAF/DLF	2D-PAGE, SRM, ELISA	nLC–MS/MS, SELDI-ToF	secretome analysis; abundant DEPs; biomarker discovery and validation	GLYCOLYSIS; COMPLEMENT; cell-stroma communication	[70,71,72,73]
Comparison of adjacent healthy/benign breast disease vs. lymph node ± IDC vs. matched LNM	FFPE (ER+/HER2- negative and matched LNM)	pulsed-SILAC assay, IHC	UHPLC-EASY spray ionization source-MS/MS	BC progression; proteomic profiles of LNM similar to those of primary tumors	proteostasis alteration: downregulation of DNA repair proteins; upregulation of ribosomal, lysosomal and proteasomal proteins; elevated rate of protein translation; deregulation of protein folding machinery; increased amounts of unfolded proteins; metabolic reprogramming: OXPHOS and GLYCOLYSIS; ROS upregulation; reduced biosynthesis and increased breakdown of fatty acids, decrease in cholesterol biosynthesis, increase in peroxisomal β-oxidation	[81]
	IDC PBT and matched LNM	2-DE	MALDI-ToF/ToF MS	overexpressed proteins in PBT	cytoskeleton reorganization, cell growth and proliferation, ECM remodeling, proteolysis regulation, metabolic reprogramming, detoxification, stress-related mechanisms, membrane-associated proteins	[80]
	serum benign, LNM+IDC and LNM-IDC	2-DE, ELISA	LC–MS/MS	DEPs during IDC progression	putative biomarkers for early metastasis detection	[82]
Comparison of IDC-stages’ specific protein signatures	serum	hydrogel nanoparticles for protein enrichment technology	LC–MS/MS	IDC early-stage proteins; LMW proteins and protein fragments as IDC candidate biomarkers	EMT [92]	[3]
	IDC PBT (mastectomy)	SDS-PAGE	LC–MS/MS	specific proteins (stage 2 and 3); identification of putative IDC stage-specific biomarkers	stage 2: proliferation, invasion, migration, stress pathways stage 3: invasion, stress, DNA repair, tumor suppression, inflammation, invasion, glycolysis, metastasis	[83]
IDC-subtype specific protein signature and biomarkers discovery	FF	2-DE, WB	LC–MS^E^, MALDI-MS/MS	IDC-specific signature of ER+/HER2/*neu* negative IDC, PPI networks	EMT; cytoskeleton organization; ROS and stress response; Calcium-binding proteins involved in signaling pathways	[23]
Comparison of IDC tumor-adjacent stroma vs. tumor-distal stroma	cell lines; FFPE	LCM, IHC	LC–MS/MS	proteomics of breast cell line-stimulated fibroblast ECM vs proteomics of invasive/metastatic stromal tissue	EMT	[85]
IDC matrisome-targeted proteomics	FF FFPE	TPM, SHG, IF	LC–MS/MS	ECM proteomic profile as early diagnosis and risk of metastases biomarker and therapeutic target	ECM remodeling: collagen fiber reorganization/alignment	[87]
	FFPE	TMA, microscopy	MALDI-FT-ICR MSI; HRAM nanoLC-ESI–MS/MS	alteration of multiple collagen patterns in TME	EMT-related biomarkers [93]	[94]
Discovery of IDC candidate biomarkers	urine, cell lines, FFPE	WB, IHC	LC–MS/MS	DEPs, biomarker candidates for early detection	acute phase response signaling, production of NO and ROS in macrophages, IL-12 signaling and production in macrophages, intrinsic prothrombin activation pathway, clathrin-mediated endocytosis signaling, communication between innate and adaptive immune cells	[74]
Pathway analysis and biomarker discovery	bioinformatics approach			proteins expression profile, prognostic significance	COAGULATION; EMT; ANGIOGENESIS; UV_RESPONSE_DN; TGF_BETA_SIGNALING; HEDGEHOG_SIGNALING	[89]

Abbreviations: AJ—apical junction; BP—biological processes; CAFs—cancer associated fibroblasts; 2-DE—two dimensional gel electrophoresis; DEPs—differentially expressed proteins; DIGE—difference gel electrophoresis; DLF—ductal lavage fluid; ECM—extracellular matrix; ELISA—enzyme-linked immunosorbent assay; EMT—epithelial–mesenchymal transition; ER—estrogen receptor; FC—flow cytometry; FF—fresh frozen; FFPE—formalin fixed, paraffin-embedded; H&E—hematoxylin and eosin stain; HPLC–MS/MS—high performance liquid chromatography coupled with tandem mass spectrometry; HER2—receptor tyrosine-protein kinase erbB2; HRAM—high-resolution, accurate-mass spectrometry; ILC—invasive lobular carcinoma; IDC—invasive ductal carcinoma; IDC-NST—invasive ductal carcinoma no special type; IF—immunofluorescence; IHC—immunohistochemistry; IL-12—interleukin 12; HNs—core-shell hydrogel nanoparticles; LC-ESI–MS/MS—liquid chromatography-electrospray ionization tandem mass spectrometry; LC-MS^E^—liquid chromatography mass spectrometry in data-independent analysis mode; LC-MS/MS—liquid chromatography tandem mass spectrometry; LCM—laser capture microdissection; LMW—low-molecular-weight proteins; LNM—lymph node metastasis; MALDI-FT-ICR-MS—matrix-assisted laser desorption/ionization Fourier transform ion cyclotron resonance mass spectrometry; MALDI-MS/MS—matrix-assisted laser desorption/ionization tandem mass spectrometry; MALDI-ToF/ToF MS—matrix-assisted laser desorption/ionization time-of-flight tandem mass spectrometry; MMP-2/9—matrix metalloproteinases 2/9; MudPIT—multidimensional protein identification technology; NAF—nipple aspirate fluid; NO—nitric oxide; OXPHOS—oxidative phosphorylation; PBT—primary breast tumor; PPI—protein–protein interactions; ROS—reactive oxygen species; SDS-PAGE—sodium dodecyl-sulfate polyacrylamide gel electrophoresis; SELDI—surface-enhanced laser desorption–ionization; SILAC—stable isotope labeling with amino acids in cell culture; SHG—second-harmonic generation microscopy; SRM—selected reaction monitoring; TMA—tissue microarray; TME—tumor microenvironment; TmT—tandem mass tag; TPM—two-photon microscopy; ToF MS—time-of-flight mass spectrometry; WB—Western blotting.

**Table 2:** IDC-dysregulated proteins involved in cytoskeletal function, adhesion, and EMT.

Dysregulated Proteins	Genes	Proteomics-Based Methods	Functions	Associated Roles in Cancer	References
Actin isoforms	*ACTB*, *ACTG*	LC–MS^E^, MALDI-MS/MS	cytoskeleton structural protein	cell growth, migration, invasion, metastasis [98], EMT [99]	[23]
Tubulin isoforms	*TUBB*, *TUBA1A*, *TUBA1B*	MALDI-ToF/ToF MS	constituents of microtubules	chromosome segregation during mitosis [100]	[84]
Keratins	*KRT19*, *KRT8*	MALDI-ToF/ToF MS	cytoplasmic intermediate filament proteins	tumorigenic transformation of cells, stemness, cell proliferation, migration [101]; EMT [102]	[80]
Vimentin	*VIM*	IHC; TmT, HPLC–MS/MS + MudPIT; LC–MS^E^, MALDI-MS/MS; MALDI-ToF/ToF MS	cytoplasmic intermediate filament protein	EMT [103]	[23,65]
Filamins	*FLNA*	nanoHPLC–MS/MS; HNs coupled with LC–MS/MS	actin-binding protein	cancer progression, cell motility, EMT [104]	[3,105]
Tropomyosin family	*TPM3*, *TPM4*	salivary LC–MS/MS; MALDI-ToF/ToF MS	actin-binding protein	cell migration, invasion, motility, metastasis, EMT [106]	[75,84]
Profilin family	*PFN1*	salivary LC–MS/MS; LC–MS^E^; MALDI-MS/MS	actin-binding protein	cell proliferation, motility, EMT [107]	[23,75]
Gelsolin	*GSN*	salivary LC–MS/MS	actin-binding protein	cell motility, EMT [108]	[75]
Cofilin	*CFL1*	serum HNs coupled with LC–MS/MS	actin-binding protein	cytoskeletal reorganization, lamellipodium formation, EMT [109]	[3]
Transgelin	*TAGLN*	LC–MS^E^; MALDI-MS/MS	actin-binding protein	cell growth, ECM degradation, invasion, metastasis, proliferation, EMT [110]	[23,85]
Ezrin	*EZR*	salivary LC–MS/MS	membrane-cytoskeleton linker	cytoskeleton remodeling, EMT [111]	[75]
Integrins	*ITGA2B*	serum HNs coupled with LC–MS/MS	membrane adhesion receptors	adhesion, recognition, immune response, cell growth, metastasis [112]	[3]
Talin	*TLN1*	serum HNs coupled with LC–MS/MS	component of adhesion complexes	cell migration, adhesion, integrin signaling [113]; EMT [114]	[3]

Abbreviations: ACTB—actin beta; ACTG—gamma actin; CFL1—cofilin 1; EMT—epithelial-mesenchymal transition; EZR—ezrin; FLNA—filamin A; GSN—gelsolin; HNs-core—shell hydrogel nanoparticles; ITGA2B—integrin A2B; KRT8—keratin 8; KRT19—keratin 19; LC-MS^E^—liquid chromatography mass spectrometry in data-independent analysis mode; LC-MS/MS—liquid chromatography tandem mass spectrometry; MALDI-MS/MS—matrix-assisted laser desorption/ionization tandem mass spectrometry; MALDI-ToF/ToF MS—matrix-assisted laser desorption/ionization time-of-flight tandem mass spectrometry; MudPIT—multidimensional protein identification technology; PFN1—profilin 1; TAGLN—transgelin; TLN1—talin 1; TmT—tandem mass tag, TPM3—tropomyosin 3; TPM4—tropomyosin 4; TUBA1A—tubulin alpha 1a; TUBA1B—tubulin alpha 1b; TUBB—beta tubulin; VIM—vimentin.

**Table 4:** Proteomics-based investigation of protein isoforms in IDC.

Protein Isoforms	Biological Samples	Other Conventional Analytical and Coupled Methods	MS-Based Proteomics	Results	Functions	References
Folate receptor isoforms (FRα, FRβ); potential isoform-based diagnosis in BC	BC cells lines and IDC tissue	WB, IHC	LC-ESI–MS/MS	simultaneous and accurate quantification of FR isoforms: FRα is overexpressed in BC cells and tissue samples, FRβ is abundant in TAMs	uni-directional folate transport into cells	[27]
Progesterone receptor isoforms A and B; PRA/PRB ratios during BC progression	BC cell line model	SDS-PAGE	HPLC–MS/MS	isoform-specific changes in BC proteome; high PRA/PRB ratios in BC associated with resistance to chemotherapy and poor prognosis	cell metabolism, cell cycle, apoptosis	[157]
Haptoglobin and α1-AT precursor isoforms	serum	2-DE; FFPE tissue sections-IHC	MALDI-MS	DEPs; identification of novel serum biomarkers in IDC patients compared with healthy women	possible role in tumor growth	[67]
Alternative splicing of ceramide synthase 2 (AS CERS2)	BC cell lines, IDC and adjacent normal tissue	RT-PCR, WB, SDS-PAGE, IHC	LC–MS/MS	higher expression of AS CERS2 in luminal B IDC	dysregulation of sphingolipid pathway, cancer initiation, proliferation and migration, cell survival, apoptosis	[160]

Abbreviations: BC—breast cancer; 2-DE—two dimensional gel electrophoresis; DEPs—differentially expressed proteins; FFPE—formalin-fixed paraffin-embedded; FRα—folate receptor alpha; FRβ—folate receptor beta; HPLC–MS/MS—high performance liquid chromatography coupled with tandem mass spectrometry; IDC—invasive ductal carcinoma; IHC—immunohistochemistry; LC-ESI–MS/MS—liquid chromatography-electrospray ionization tandem mass spectrometry; LC-MS/MS—liquid chromatography tandem mass spectrometry; MALDI-MS—matrix-assisted laser desorption/ionization mass spectrometry; MS—mass spectrometry; PRA—progesterone receptor isoform A; PRB—progesterone receptor isoform B; SDS-PAGE—sodium dodecyl-sulfate polyacrylamide gel electrophoresis; WB—Western blotting.

## Data Availability

Not applicable.
